# Bioinformatic Analysis of Gene Variants from Gastroschisis Recurrence Identifies Multiple Novel Pathogenetic Pathways: Implication for the Closure of the Ventral Body Wall

**DOI:** 10.3390/ijms20092295

**Published:** 2019-05-09

**Authors:** Víctor M. Salinas-Torres, Hugo L. Gallardo-Blanco, Rafael A. Salinas-Torres, Ricardo M. Cerda-Flores, José J. Lugo-Trampe, Daniel Z. Villarreal-Martínez, Laura E. Martínez de Villarreal

**Affiliations:** 1 School of Medicine and University Hospital “Dr. José Eleuterio González”, Department of Genetics, Universidad Autónoma de Nuevo León, Ave. Madero y Gonzalitos S/N Col. Mitras Centro, Monterrey CP 64460, Mexico; lugotramjose@hotmail.com (J.J.L.-T.); zaca_2695@hotmail.com (D.Z.V.-M.); laelmar@yahoo.com.mx (L.E.M.d.V.); 2 Instituto Tecnológico de Tijuana, Department of Systems and Computing, Calzada del Tecnológico S/N Fracc, Tomas Aquino, Tijuana CP 22414, Mexico; rafaelsalinas.itt@gmail.com; 3 School of Nursing, Universidad Autónoma de Nuevo León, Dr. José Eleuterio González 1500, Mitras Centro, Monterrey CP 64460, Mexico; ricardocerda_mx@yahoo.com.mx

**Keywords:** abdominal wall defect, alleles, bioinformatics, development, gastroschisis, genes, genetics, pathogenesis, recurrence, whole exome sequencing

## Abstract

We investigated whether likely pathogenic variants co-segregating with gastroschisis through a family-based approach using bioinformatic analyses were implicated in body wall closure. Gene Ontology (GO)/Panther functional enrichment and protein-protein interaction analysis by String identified several biological networks of highly connected genes in *UGT1A3, UGT1A4, UGT1A5, UGT1A6, UGT1A7, UGT1A8, UGT1A9, UGT1A10, AOX1, NOTCH1, HIST1H2BB, RPS3, THBS1, ADCY9*, and *FGFR4*. SVS–PhoRank identified a dominant model in *OR10G4* (also as heterozygous de novo), *ITIH3, PLEKHG4B*, *SLC9A3*, *ITGA2*, *AOX1*, and *ALPP*, including a recessive model in *UGT1A7*, *UGT1A6*, *PER2*, *PTPRD*, and *UGT1A3*. A heterozygous compound model was observed in *CDYL, KDM5A*, *RASGRP1*, *MYBPC2*, *PDE4DIP*, *F5*, *OBSCN*, and *UGT1A*. These genes were implicated in pathogenetic pathways involving the following GO related categories: xenobiotic, regulation of metabolic process, regulation of cell adhesion, regulation of gene expression, inflammatory response, regulation of vascular development, keratinization, left-right symmetry, epigenetic, ubiquitination, and regulation of protein synthesis. Multiple background modifiers interacting with disease-relevant pathways may regulate gastroschisis susceptibility. Based in our findings and considering the plausibility of the biological pattern of mechanisms and gene network modeling, we suggest that the gastroschisis developmental process may be the consequence of several well-orchestrated biological and molecular mechanisms which could be interacting with gastroschisis predispositions within the first ten weeks of development.

## 1. Introduction

Gastroschisis constitutes one the leading categories of human birth defects concerning the ventral body wall development [1]. It is characterized by the evisceration of abdominal organs, usually to the right of the umbilical ring, lacking a protective membrane. Emerging evidence suggests that gastroschisis (an epidemiologic and pathogenetic dilemma) could be the consequence of a complex interplay of functionally interacting genetic and non-genetic factors as precipitating elements in its pathogenesis [2,3,4]. These influences have shown to be involved in several crucial biological processes such as blood vessel and epithelium development, cell adhesion, regulation of cytokine biosynthetic process, and regulation of developmental gene expression [2,3,4].

Studying biological processes and pathways through bioinformatic analysis in gastroschisis may have an important clinical relevance. Pregnancies affected with fetal gastroschisis can have multiple complications such as low fetal weight, in utero fetal demise, or preterm delivery [1]. Since this defect develops early in gestation, it is possible that alterations in the regulation of embryonic or fetal vascular, epithelium, immune development, or gene expression may play a role in these related problems [4]. Moreover, it may provide plausible clues to the closure of the ventral body wall.

Given the current paucity of genes with causal implication in human gastroschisis, we leveraged on genes and DNA variations co-segregating with gastroschisis through a family-based approach identified by whole exome sequencing (WES) [4], and investigated whether likely pathogenic variants were implicated in body wall closure through bioinformatic analysis to explore potential pathogenetic pathways with this disease.

## 2. Results

A total of 429 genes involving heterozygous DNA variations co-segregating with gastroschisis have been identified and selected from the WES gene list for further bioinformatic analyses [4].

### 2.1. Gene Functional Enrichment Analysis 

Gene Ontology (GO) Consortium and Panther Classification System databases [5] identified several highly enriched GO terms (False Discovery Rates, FDR) within genes co-segregating in the family with recurrence for gastroschisis (Table 1).

Functional biological processes related to xenobiotic glucuronidation, negative regulation of glucuronosyltransferase activity, and flavonoid glucuronidation were the most significantly enriched terms (Subfamily *UGT1A* involving *UGT1A3, UGT1A4, UGT1A5, UGT1A6, UGT1A7, UGT1A8, UGT1A9, UGT1A10, P* FDR 7.99 × 10^−11^, 1.42 × 10^−9^, and 2.26 × 10^−7^, respectively). 

Cellular component GO analysis also revealed the integral component of membrane as the most significant functional category (*UGT1A3, UGT1A4, UGT1A5, UGT1A6, UGT1A7, UGT1A8, UGT1A9, UGT1A10, AOX1, NAA30, OMA1, DPYD, PDIA2, ALPP, CDYL, ADPGK, KDM5A, PRDM9, ZFHX3, BCAS3, NOTCH1, TELO2, PLEKHG4B, SPATA17, LRP5, AXIN1, PKD1, SLC9A3, GAL3ST3, RASGRP1, AKAP6, HIST1H2BB, EIF3L, RPS3, RPS6, MRPS6, INPP5B, IRS1, FGFR4, FGFRL1, EFNA5, ATP13A5, TBX21, TLR8, ADCY9, GRM4, GRIN2C, OR13F1, OR10G4, OR4C3, OR2C1, F5, THBS1, MAP2K3, RTN3, EVPL, KLK14, ITGA7, ITGA2B, ITGA2, ITGAD, CTCFL*, and *RABL6, P* FDR 4.95 × 10^−3^). 

The molecular function resulted mainly enriched in glucuronosyltransferase activity (*UGT1A, P* FDR 1.58 × 10^−6^), whereas KEGG signaling pathway analysis identified drug metabolism—other enzymes and pentose and glucuronate interconversions, as the most significant functional categories (*UGT1A* and *AOX1, P* FDR 1.32 × 10^−8^ and 2.83 × 10^−8^, respectively).

### 2.2. Protein-Protein Interactions Network Analysis 

**Figure 1:**
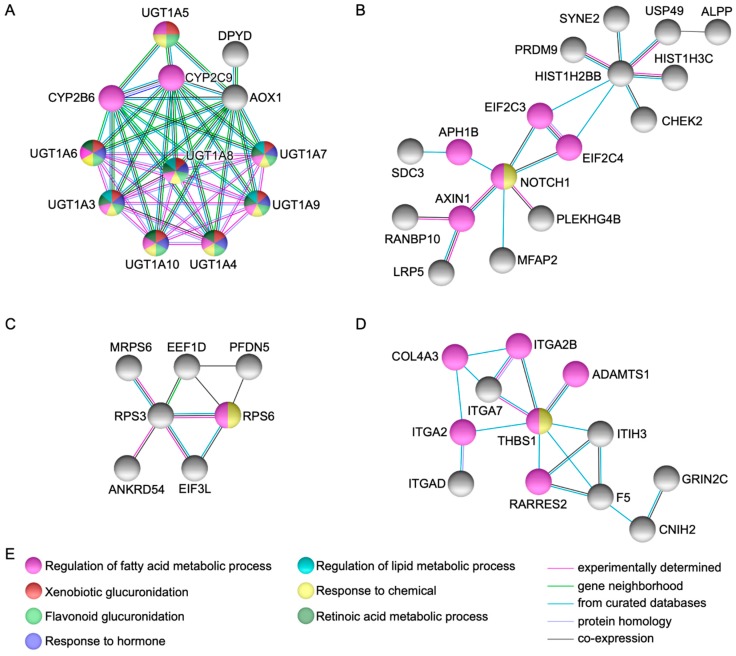
Network modeling from protein-protein interactions. The figures (**A**)–(**D**) show high connectivity and direct protein-protein interactions via several partners in genes *UGT1A3*, *UGT1A4*, *UGT1A5*, *UGT1A6*, *UGT1A7*, *UGT1A8*, *UGT1A9*, *UGT1A10*, *AOX1*, *NOTCH1*, *HIST1H2BB*, *RPS3*, and *THBS1*. Figure (**E**) depicts the biological processes identified in the networks.

**Figure 2:**
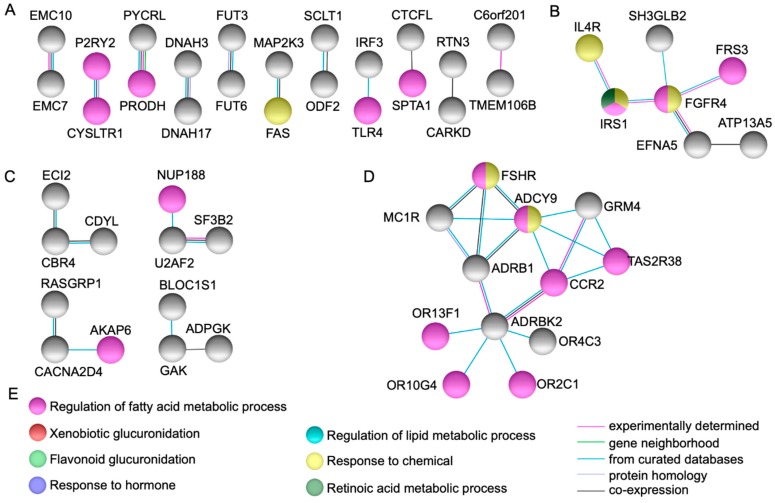
Network modeling from protein-protein interactions. Figures (**A**) and (**C**) show several direct protein-protein dyads and triads. Figures (**B**) and (**D**) show high connectivity and direct protein-protein interactions via several partners in genes *ADCY9* and *FGFR4*. Figure (**E**) depicts the biological processes identified in the networks.

String protein-protein interaction analysis [6] identified fifteen genes represented by *UGT1A3, UGT1A4, UGT1A5, UGT1A6, UGT1A7, UGT1A8, UGT1A9, UGT1A10, AOX1, NOTCH1, HIST1H2BB, RPS3, THBS1, ADCY9*, and *FGFR4*, that showed high connectivity to each other and to other genes in the network via several partners (Figure 1 and Figure 2).

Several protein-protein dyads and triads were observed as depicted in Figure 2. Of note, *FGFR4* (p.G388R) (rs351855) was a coding non-synonymous variant identified in the index case and both parents [4].

### 2.3. Classification by Inheritance Pattern

SVS–PhoRank gene ranking [7] classified the inheritance pattern for all variants (Tables S1–S3). A dominant model score was noted for the following variants: *OR10G4* (rs4474449, also as heterozygous de novo variant), *ITIH3* (rs2286797), *PLEKHG4B* (rs114885385), *SLC9A3* (rs117027344), *ITGA2* (rs1062535), *AOX1* (rs3731722), and *ALPP* (rs13026692). Furthermore, *UGT1A7* (rs11692021), *UGT1A6* (rs6759892, rs2070959, rs1105879), *PER2* (rs934945), *PTPRD* (rs3824417), and *UGT1A3* (rs6431625) were identified within a recessive model score as well as homozygous both.

**Figure 3:**
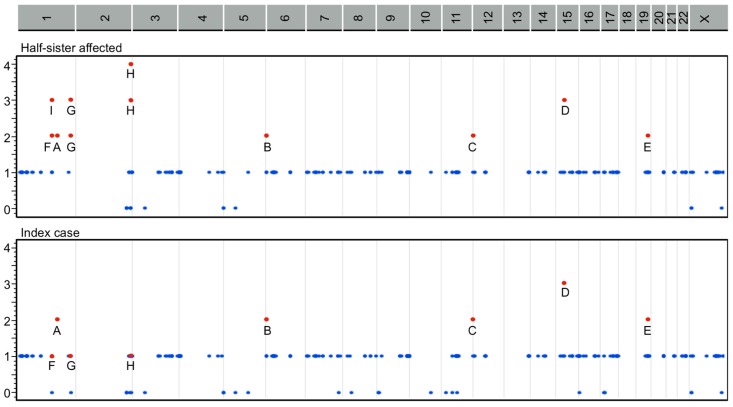
Heterozygous compound model score regions from gene variants identified in *F5* (**A**), *CDYL* (**B**), *KDM5A* (**C**), *RASGRP1* (**D**), *MYBPC2* (**E**), *PDE4DIP* (**F**), *OBSCN* (**G**), *UGT1A* (**H**), and 1:144146811–146467744 chromosomal region including *PDE4DIP* (**I**) among both cases and the mother (significant heterozygous regions as red points). Vertical scales list the number of heterozygous genotypes inherited from the mother. Horizontal scales depict the chromosomal region involved.

A heterozygous compound model score was observed in the following variants among the mother and both cases: *CDYL* (rs11965336 and rs11962921), *KDM5A* (rs148769146 and rs144276601), *RASGRP1* (rs56241040, rs55704435, and rs56366451), *MYBPC2* (rs75197332 and rs187765595), *PDE4DIP* (rs61804988, rs144590426, and rs2762779), *F5* (rs6027 and rs1800595), *OBSCN* (rs75280352, rs185523702, and rs72762068), and *UGT1A* (rs11692021, rs6759892, rs1105879 homozygous genotype in the index case, and rs6431625 homozygous genotype in the half-sister affected) (Figure 3).

### 2.4. Pathogenetic Pathways from Selected Genes Co-Segregating with Gastroschisis

According to ToppGene Suite [8], eleven pathogenetic pathways were identified based on manually curated GO categories among selected genes co-segregating in the family with recurrence for gastroschisis (Table 2).

There was no data available within GO functional categories for *SPATA17* and *CFAP65*. Table S4 includes the complete result list of GO biological processes and pathways.

## 3. Discussion

In the present study, we employed bioinformatic analyses using genes and genetic variations co-segregating with gastroschisis through a family-based approach identified by WES as a model system, to explore potential pathways implicated in body wall closure. Our findings demonstrate that several functional GO categories were displaying plausibility (Table 1, Figure 1 and Figure 2), whereas novel gene variants within dominant, recessive, and heterozygous compound models (Tables S1–S3, Figure 3) were possibly underlying to gastroschisis as well. We also characterized multiple novel pathogenetic pathways that may have meaningful consequences for the closure of the ventral body wall in gastroschisis (Table 2 and Table S4).

A limitation of the study was that a different filter from bioinformatics tools could prioritize some gene variants and hence, cause their inclusion or exclusion. Thus, findings from bioinformatic algorithms can only be taken as suggestive evidence in the absence of functional validation. However, it must be noted that our results were validated from independent bioinformatics platforms, giving the basis for further validation and reproducibility as well as to obtain more information about the molecular mechanisms involved in gastroschisis.

### 3.1. Novel Candidate Genes and Pathogenetic Pathways

Several genes could be potential candidates for future studies as those observed with a highly significant functional enrichment, high connectivity/direct protein-protein interactions, and highest score for dominant, recessive, and heterozygous compound models (*UGT1A3, UGT1A4, UGT1A5, UGT1A6, UGT1A7, UGT1A8, UGT1A9, UGT1A10, AOX1, NOTCH1, HIST1H2BB, RPS3, THBS1, ADCY9, FGFR4, OR10G4*, *ITIH3*, *PLEKHG4B*, *SLC9A3*, *ITGA2*, *ALPP, PER2*, *PTPRD*, *CDYL*, *KDM5A*, *RASGRP1*, *MYBPC2*, *PDE4DIP*, *F5*, and *OBSCN*) (Table 1 and Tables S1–S3, Figure 1, Figure 2 and Figure 3). 

High protein-protein interactions as well as functional enrichment were observed in xenobiotic genes UDP-glucuronosyltransferases (*UGT1A* at 2q37.1, heterozygous compound model) in *UGT1A3* (recessive model and moderate impact), *UGT1A4* and *UGT1A5* (modifier impact), *UGT1A6* (recessive model, moderate and modifier impact), *UGT1A7* (recessive model and moderate impact), *UGT1A8*, *UGT1A9*, and *UGT1A10* (modifier impact). UDPGTs bind to enzyme, carbohydrate, and protein conjugation with subsequent deactivation and elimination of potentially toxic xenobiotics (e.g., drugs, environmental pollutants, dietary chemicals) and endogenous compounds (e.g., bilirubin, steroids, bile acids). Moreover, *CYP2C8* along with *CYP2C9* and *CYP2B6* via cytochrome P450-mediated oxidation may undergo glucuronidation activities (Figure 1A) [9,10]. UDPGTs have been associated with a variety of bilirubin metabolic disorders and their mouse orthologs *Ugt1a1, Ugt1a2, Ugt1a5, Ugt1a6b, Ugt1a7c, Ugt1a8, Ugt1a9, Ugt1a10* are mainly expressed in the liver at days 15.5 and 18.5 (Carnegie stages); corresponding to the 6th and 7th week of development in humans (WD, weeks from fertilization) [9,11,12].

*AOX1* (Aldehyde Oxidase 1 at 2q33.1, dominant model, moderate and modifier impact), was connected directly and via partners with *UGT1A, CYP2C9, CYP2B6*, and *DPYD*; the latter, (moderate and modifier impact) is related to pyrimidine metabolism and nucleotide synthesis. *AOX1* is involved in the regulation of reactive oxygen species homeostasis and has been associated to hereditary xanthinuria [9]. The mouse ortholog *Aox1* was found expressed in the liver at day 15.5 (6th WD) [12].

Further protein-protein interactions were noted in *NOTCH1* (Notch 1 at 9q34.3, modifier impact), *PLEKHG4B* (Pleckstrin Homology and RhoGEF Domain Containing G4B at 5p15.33, dominant model and modifier impact), and *HIST1H2BB* (Histone Cluster 1 H2B Family Member B at 6p22.2, modifier impact) (Figure 1B). GO molecular annotations for these genes implies gene expression, Rho guanyl-nucleotide exchange factor activity, core promoter binding, as well as chromatin, DNA, and protein binding [9,11]. Noticeably, *Notch1* can be expressed both in trophoblast and embryonic stem cells from preimplantation through all stages of development [9,13], whereas *Hist1h2bb* was found expressed in the liver and brain between days 15.5 and 18.5 (6th and 7th WD) [12]. Of interest, *KDM5A* (Lysine Demethylase 5A at 12p13.33) and *CDYL* (Chromodomain Y Like at 6p25.1) both as heterozygous compound model and modifier impact genes, were closely interacting to this particular network. These genes play a central role in histone code, regulating specific gene transcription and repression of essential biological processes preserving the epigenetic landscape [9]. The mouse orthologs *Kdm5a* and *Cdyl* were found expressed in the cell stage embryo, blastomere, and blastocyst (days 1.5 to 4.5, 1st WD) [14,15] as well as in the liver and brain at days 15.5 and 18.5 (6th and 7th WD) [12].

*RPS3* (Ribosomal Protein S3 at 11q13.4, modifier impact) was detected interacting with multiple genes implicated in the regulation of protein synthesis (Figure 1C). This gene plays a key role in the protection of DNA, binding with similar affinity to intact and damaged DNA, and when located in the mitochondrion, reduces cellular reactive oxygen species levels and mitochondrial DNA damage. Moreover, it binds and protects *TP53/p53* from *MDM2*-mediated ubiquitination and is involved in the induction of apoptosis through its role in the activation of *CASP8* [9]. *Rps3* was detected in the liver and brain at day 15.5 (6th WD) [12].

Additional direct and via partners protein-protein interactions were observed in *THBS1* (Thrombospondin 1 at 15q14, moderate and modifier impact), *ITGA2* (Integrin Subunit Alpha 2 at 5q11.2, dominant model and modifier impact), *ITIH3* (Inter-Alpha-Trypsin Inhibitor Heavy Chain 3 at 3p21.1, dominant model and modifier impact), and *F5* (Coagulation Factor V at 1q24.2, heterozygous compound model, moderate and modifier impact) (Figure 1D). These genes have been associated with a variety of thrombotic abnormalities, as they play a central role in hemostasis and cell adhesion, mediating and stabilizing the adhesion of platelets (including other cell types such as collagen) to the extracellular matrix [9]. Expression analysis of mouse *Thbs1* was identified in the yolk sac and embryo mesenchyme at days 9.5 and 14.5 (4th and 5th WD), respectively [16,17], whereas *Itga2* was found expressed in the embryo, epidermis, blood vessel endothelium, and epidermis stratum basale at days 7.5, 16.5, and 18.5 (3rd, 6th, and 7th WD), respectively [18,19]. *Itih3* was found expressed in the liver at day 15.5 (6th WD) [12] and *F5* was detected in the embryo and notochord at days 7.5 to 14.5 (3rd to 5th WD) and 8.0 to 8.5 (3rd WD), respectively [20,21].

*ADCY9* (Adenylate Cyclase 9 at 16p13.3, modifier impact) was noted to interact with multiple genes implicated in G protein-coupled receptor activities, including catecholamine-induced activation, chemotaxis and migration, follitropin, melanogenesis, and signal transduction of odorant and taste molecules (Figure 2D). This gene contributes to signaling cascades activated by the corticotropin-releasing factor, corticosteroids, and beta-adrenergic receptors, implicated in the production of platelet-derived and vascular endothelial growth factors [9]. Murine *Adcy9* was detected in the cardiovascular system, axial skeleton, skin, and hair at day 14.5 (5th WD) [22].

*FGFR4* (Fibroblast Growth Factor Receptor 4 at 5q35.2) was observed interacting with several genes involved in the regulation of tyrosine kinases activities and cell surface receptors for fibroblast growth factors (Figure 2B). These genes are crucial for diverse developmental, metabolic, immune, and cancer processes, as they regulate cell proliferation, differentiation, and migration, including the regulation of lipid metabolism, bile acid biosynthesis, glucose uptake, vitamin D metabolism, and phosphate homeostasis [9]. Murine *Fgfr4* was detected in the cell stage embryo and blastocyst at days 2 and 3.5, respectively (1st WD) [15,23], as well as in the neural ectoderm, embryo mesenchyme, extra embryonic ectoderm at day 7.5 (3rd WD) and the somite, yolk sac endoderm, and endoderm of the developing gut from days 8.5 to 14.5 (3rd to 5th WD) [24,25]. Moreover, it became restricted at low levels of expression to the liver and the central nervous system from day 11.5 (4th WD) onward [23].

Bioinformatic classification by inheritance pattern also identified *PER2* (Period Circadian Regulator 2 at 2q37.3, recessive model and moderate impact). This gene regulates circadian rhythms in gene expression, which are translated into rhythms in metabolism and behavior, including a wide array of physiological functions such as sleep, body temperature, blood pressure, endocrine, immune, cardiovascular, and renal functions [9]. *Per2* was identified in the future spinal cord and central nervous system at days 11.5 and 13.5 (4th WD) [26,27], the blood vessel and cardiovascular system at day 14.5 (5th WD) [22], and the liver at day 15.5 (6th WD) [12].

*SLC9A3* (Solute Carrier family 9 member A3 at 5p15.33, dominant model and modifier impact), is an epithelial brush border Na/H exchanger that eliminates acid-toxicity generated by active metabolism or counters adverse environmental conditions [9]. The mouse ortholog *Slc9a3* was found expressed from the cell stage embryo to trophectoderm (days 1.0 to 3.5, 1st WD) [28,29] as well as in the metanephros at day 18.5 (7th WD) [30].

*RASGRP1* (RAS Guanyl Releasing Protein 1 at 15q14, heterozygous compound model and modifier impact) and *PTPRD* (Protein Tyrosine Phosphatase Receptor type D at 9p24.1-p23, recessive model and modifier impact) are crucial signaling molecules critical for a variety of cellular processes including cell growth, differentiation, mitotic cycle, and oncogenic transformation, by regulating T-cell/B-cell development, NK cell cytotoxicity, and ITAM-dependent cytokine production by activation of Ras-mediated ERK/MAPK and JNK pathways [9]. Mouse orthologs *Rasgrp1* and *Ptprd* were detected in the olfactory lobe/epithelium at day 14.5 (5th WD) as well as in the liver and central nervous system at days 15.5 and 16.5 (6th WD) [12,17,31].

*MYBPC2* (Myosin Binding Protein C, Fast Type at 19q13.33, heterozygous compound model and modifier impact) and *OBSCN* (Obscurin at 1q42.13, heterozygous compound model, moderate and modifier impact) are key signaling proteins that play a role in the regulation of myofibrillogenesis and modulation of muscle contraction, as they bind and modify the assembly of actin-activated myosin ATPase into sarcomeric A bands in striated muscle [9]. Murine *Mybpc2* was found expressed in the hindlimb at day 18 (7th WD) [32], whereas *Obscn* was detected in the diaphragm and cardiomyocytes at days 14.5 and 17 (5th and 6th WD), respectively [17,33].

Overall, these interacting genes (together with previous high impact variants and gene variants segregating among both cases and the mother) [4], demonstrated significant representation of xenobiotic, regulation of metabolic processes, regulation of cell adhesion, regulation of gene expression, inflammatory response, regulation of vascular development, keratinization, left-right symmetry, epigenetic, ubiquitination, and regulation of protein synthesis related GO categories (Table 2 and Table S4). Thus, based on our results, multiple novel pathogenetic pathways could be implicated in gastroschisis pathogenesis. Indeed, our findings point to a complex interplay of functionally interacting genetic and non-genetic factors; thereby, we propose that each of these interacting genes are likely substantial influences to its development, underscoring major gaps concerning the current knowledge of genetics and developmental biology of the defect.

### 3.2. Theories on the Failure of Body Wall Closure in Human and its Implication for Gastroschisis

As enlisted in Table S2, a considerable number of GO categories evoking related pathogenetic theories in gastroschisis were identified [34,35,36,37,38,39,40,41,42,43]. These hypotheses along with our proposed “multigenic/multifactorial model” [4], highlight novel susceptibility gene variants within pathogenetic pathways that could be possibly implicated in the pathogenesis of gastroschisis (Table 2).

Perhaps the chief explanation for an experimental scarcity regarding the biological or molecular mechanism of human ventral body wall development, particularly in gastroschisis, is linked to the assumption that such defect does not have a genetic basis. Recently, we published research suggesting that this phenotype may result from complex gene-gene or gene-environment interactions [1,2,3,4,44,45]. Accordingly, it is possible that several of the above pathogenetic pathways do indeed lead to a disruptive closure of the ventral body wall, as several well-orchestrated biological and molecular mechanisms could be interacting with gastroschisis genetic predisposition within the first ten weeks of human development. 

Previous reports from ortholog expression tissues assays involving the above-described genes indicated that reduced but significant gene predispositions (as those implicated in the regulation of gene expression) are expressed in cell-embryo and implantation stages [13,14,15,22,23,28,29], whereas several genetic influences implicated in the regulation of metabolic processes, regulation of vascular development, regulation of protein synthesis, and ubiquitination may lead to a disruptive closure of the ventral body wall in a fast-growing or massive fashion presumably between the 3rd and 5th WD [13,16,17,18,20,21,22,23,24,25,26,27]. Notably, multiple factors and gene interactions (background modifiers) can aggravate or mask the expected phenotype through gain- or loss-of-function mutations in genes or gene pathways involved in related disease-relevant cells [46,47,48], perhaps giving an isolated anomaly. In these stages, folding of the embryo due to a rapid increase in the proliferation of the neuroectoderm and the underlying mesoderm occurs, giving rise to the four folds of the abdominal ventral body wall as well as the thoracic, sternum, diaphragm, and cloacal membrane. These morphogenetic events are determined due to growth arrest caused by cell death at the umbilical ring [41,49,50,51]. 

On the other hand, predominant and significant gene predispositions (as those implicated in xenobiotic, regulation of metabolic processes, regulation of cell adhesion, inflammatory response, keratinization, and epigenetic) may lead to a disruptive closure of the ventral body wall as an enlarged umbilical ring presumably between 6th and 10th WD [12,13,19,23,30,31,32,33]. Between these stages, the umbilical ring emerges from the four body folds and is located in the center of the abdomen (ventral surface) performing proper transition between the body wall and the amnion, whereas the latter remains in continuity with the periderm throughout the embryonic period. During the 10th and 11th WD, the epithelial tissues from either side of the embryo meet and fuse at the midline. Concurrently, an intermediate layer rises from the stratum basale/germinativum including collagenous and elastic fibers (periderm keratinizes and desquamates); whereas cell death and cell deposition prevents the growth of the umbilical ring maintaining approximately the same diameter throughout development [41,49,50,51,52].

Our results contribute to the growing evidence linking gastroschisis to the embryonic dysgenesis theory [3,41,49,50,53]. Aberration on the ectodermal placodes leads to disturbances in the cell deposition process and hence, the mesoderm of the ventral body wall could be underdeveloped resulting in an enlarged umbilical ring (mesodermal deficiency) [41,49,50]. An ectodermal placode expressly at the umbilical ring involves pivotal signaling either to proper transition between the amnion and the umbilical ring or to cell-cell communication from the ectoderm to the mesoderm, depositing mesoectodermal cells contributing to the lateral and ventral body wall of the embryo. Moreover, due to a combined mechanism of apoptosis and cell proliferation of the underlying ectodermal cells, this cell deposition process can be added into the mesodermal compartment that lies below, including tissues associated with ventral body wall closure [41,49,50,51]. Although significant differences take place featuring the human and mouse embryology [51], these molecular mechanisms have been successfully elucidated in rat embryos cultured in vivo and *AP-2α* knockout mouse [54,55].

Taken together, our findings favors the recently proposed “mediolateral growth in the dorsolateral body wall” as well as the “differential growth” models [56,57]. According to these models, cell migration is unnecessary for the development of new structures, as a robust increase in body size of the experimented embryos contrasted with minimal changes in size of the umbilical ring and diastasis of the rectus muscles observed from the 6th to 10th WD. These observations harbor the hypothesis that though bones and ventral body wall muscles may have undergone normal differentiation, ventral midline defects could originate from an insufficient dorsoventral growth [56]. In this sense, the development of the ventral body wall is consistent with a variety of orchestrated interactions involving a balanced adhesome phenomena for which conserved cell-matrix adhesions in vertebrate muscle morphogenesis are paramount [58,59]. Although the type of defect would depend on the degree and location of the insufficiency, such a period is consistent with the absence of associated anomalies [3,38,53,60]. Furthermore, it must be noted that previous observations in gastroschisis support failure in normal attachment between the umbilical cord and umbilical ring [37,38,53], as well as a secondary rupture of the umbilical cord following normal attachment [60].

Alternatively, the classical “lateral somitic frontier” model hypothesizes that somitic muscle cells are patterned independently migrating or translocating into the lateral plate area, as migration of ribs and muscles through the body wall occurs in a ventral direction [61]. This model recalls other theories linked to the disruption of the basic organization of embryonic development, adverse/teratogenic embryonic environment provided by young mothers, and high concurrence risk from intrauterine vascular disruption between the 3rd to 5th WD [34,35,36,39,40,42,43,62,63]. Embryonic disruption as well as poor timing of vascular transition involves essential processes such as angiogenesis, apoptosis, and cell migration. From our perspective, this biological scenario might be demonstrative for those cases displaying a constellation of co-occurring anomalies [3,64,65], or perhaps fetal demise, particularly when associated with maternal toxic exposures, given that more than one disruptive event may occur, even after the embryonic period [66]. Of note, cell deposition may also be massively reduced between the abdominal wall and the amnion, which could lead to an extensive defect of the abdominal wall along with detrimental consequences to the abdominal organs and cord [49].

### 3.3. Gastroschisis Clinical Susceptibility

In our view, all possible genetic and non-genetic factors including their direct regulators contributing to the gastroschisis complex landscape should be considered [4]. Thus, considering our results and possible variable disease presentation [3], several complex mechanisms such as epistatic mutational effect, synergistic heterozygosity or multigenic inheritance, variants in modifier genes, or effects due to overall rare variant load, may cause enhancement or repression of the phenotype [48]. Additionally, defects in pathways causing cumulative effects could lead to susceptibility or phenotypic variability (severe/unusual or milder/isolated presentation) [46,47].

Accordingly, the reason for a prevailing association of gastroschisis with young maternal age (perhaps along with low pre-pregnancy body mass index or environmental noxious influences), could be orchestrated by genes involved in several well-synchronized biological and molecular mechanisms of xenobiotic and regulation of metabolic processes. These genes may contribute towards gastroschisis risk due to an endo- or xenobiotic, flavonoid, fatty acid, drug, hormone, carbohydrate, chemical, or lipid metabolism-related toxicity. Furthermore, they also regulate the response to growth factors, detection and response to stimulus, folate biosynthesis, as well as a negative co-regulation of skeletal muscle, fibroblasts, and subcutaneous adipose tissue [9,11]; notwithstanding that epigenetic-related influences could also be interacting with a particular plethora of non-genetic risk factors such as parental alcohol intake and cigarette smoking. This appears to be the case for an increasing proportion of affected fetuses from young mothers, which may be well considered as a vulnerable population associated with a limited optimal pre/peri-conceptional care, as well as estrogen-related disruptors (decreased maternal age, nulliparity, decreased body mass, alcohol intake) [1,43].

Given the above, it follows that genes involved in the inflammatory response may play a key role for maternal immunological factors and possibly be responsible for susceptibility to genitourinary infections and the association of a change in sex partner with gastroschisis [44,45,67]. Altogether with the genes involved in the regulation of gene expression, epigenetic, regulation of protein synthesis, and ubiquitination, fetal immune development regulating genes may also play a central role in preterm birth and low fetal weight. In this context, patients with gastroschisis have shown significant high systemic levels of inflammatory cytokines and chemokines, including an earlier activation of CD4+ and CD8+ effector and memory T cells [68].

The mechanism/cause of prenatal death in gastroschisis is controversial and we do not offer any explanation for this related complication. Morphological studies provide support for a possible effect involving the umbilical cord compression due to extra-abdominal bowel herniation leading to hypoxia and eventually to fetal death [53,69]. Cytokine-mediated inflammatory response has been equally incriminated [70]. Further studies investigating vascular or cell adhesion phenomena as well as chronic inflammation/immune response could be valuable for understanding this detrimental consequence.

*CFAP65, CROCC, NOTCH1, AXIN1, LRP5, HHIP, PKD1, NR1H3*, and *IFT140* were identified to possibly influence the left-right symmetry of gastroschisis cases [4]. These genes may reflect an asymmetric arrest of vascular supply (3rd to 5th WD) [13,14,17,22,27,71,72,73,74,75,76], asymmetric cell deposition at the umbilical ring (6th to 10th WD) [53,74,75,76], or amniotic rupture along the umbilical cord in its pars flaccida (8th to 11th WD) [19,60,75], similar to the suggested weak point left after involution of the right umbilical vein between the 5th and 10th WD [37,39]. Interestingly, an ectopic expression of *Pitx2* in the right side of experimental embryos affected the left-right asymmetry of the heart, gut, and vitelline vein, resulting in phenotypes similar to those associated with *Shh* and *nodal* misexpression at days 10 to 16 (4th to 6th WD) [77]. These genes may also serve as critical transcription targets that mediates left-right asymmetry as well as left-to-rightward shunts between the left umbilical vein, the right hepatocardiac channel, and the right vitelline vein [78]. Furthermore, differential left-right expression of genes during embryonic and organogenesis stages may impact cell proliferation, cell-cell communication, and other morphogenetic processes of specific laterality development; particularly when an unfavorable/teratogenic embryonic environment or adverse genetic influences are involved [79].

Finally, a protective or risk factor owing to race/ethnicity has also been associated with gastroschisis, which may differ based on maternal nativity [80,81,82]. The effect of Hispanic ethnicity in the present study may add to this particular association as heritable factors and genetic susceptibility among the Hispanic population may play a role in the pathogenesis of gastroschisis [2,3,44,45]. Yet, when considering the lacking genomic research on gastroschisis, including this association, the proportion of genetic variation that has been evaluated requires the validation of these findings among additional populations along with its interracial and cultural variation.

In conclusion, we identified multiple novel pathogenetic pathways implicated in the closure of the ventral body wall from genes co-segregating with gastroschisis. Based on our findings and considering the plausibility of the biological pathogenetic pattern of mechanisms and gene network modeling, we suggest that the gastroschisis developmental process may be the consequence of several well-orchestrated biological and molecular mechanisms, which could be interacting with gastroschisis predispositions within the first ten weeks of human development. These genes highlight a role for xenobiotic, regulation of metabolic processes, regulation of cell adhesion, regulation of gene expression, inflammatory response, regulation of vascular development, keratinization, left-right symmetry, epigenetic, ubiquitination, and regulation of protein synthesis in gastroschisis pathogenesis.

## 4. Materials and Methods

### 4.1. Study Participants and Identification of Gene Variants from WES

The present study was approved by the Institutional Ethics Committee from the School of Medicine and University Hospital “Dr. José Eleuterio González”, Universidad Autónoma de Nuevo León, México (Approval: 21 September 2017, GN17-00002). Written informed consent was obtained from the parents. A Mexican family (two affected half-sisters with gastroschisis, mother, and father of the proband) was assessed for WES. Screening, identification, and data analyses of genes and gene variants have been previously described [4].

### 4.2. Gene Functional Enrichment Analysis

A functional enrichment analysis was performed using the GO Consortium and Panther Classification System databases, which contain comprehensive information on the evolution and function of protein-coding genes from 104 completely sequenced genomes [5]. A *P* value of less than 0.05 was the selection criteria for significantly enriched biological processes and pathways which include a “hierarchical view” as a structure of the most significant classifications and ontologies of the human genes and E-value statistics (FDR).

### 4.3. Gene and Protein Network Analysis

A protein-protein interaction network including physical, functional, and biological processes associated with genes co-segregating with gastroschisis was created using String database with a confidence score of 0.4 [6]. A gene-gene pairwise network was constructed using the protein-protein interactions. Gene pairs detected in two or more of the protein-protein interactions including co-expression, protein homology, curated databases, gene neighborhood, or experimentally determined data sets were selected and included in the network analysis using String database 10.5 [6].

### 4.4. Classification of Gene Variants by Inheritance Pattern

SVS–PhoRank gene ranking (Golden Helix®, Inc., Bozeman, MT, USA) was used to classify the inheritance pattern for all variants, which ranks genes based on their relevance to user-specified phenotypes as defined by GO and Human Phenotype Ontology biomedical ontologies [7]. A sum of the standard score above 1.333 was considered for dominant and recessive models, whereas a heterozygous compound model score was considered from the detection of at least two heterozygous genotypes within the same gene inherited from the mother at a minimum of two different loci (recessive model). The classification also included the following models: maternal de novo, paternal de novo, heterozygous de novo, homozygous de novo, heterozygous either, heterozygous maternal, heterozygous paternal, and homozygous both.

### 4.5. Pathogenetic Pathways from GO Functional Categories

ToppGene Suite (Cincinnati, OH, USA) [8] was used to classify the pathogenetic pathways (manually curated) based on the identification of related GO functional categories (biological processes and pathways) from selected genes co-segregating with gastroschisis (e.g., high impact variants and gene variants segregating among both cases and the mother, as well as further genes resulting from our analyses) [4]. GO terms were selected based on their proximity and plausibility to the phenotype, including previous pathways associated to gastroschisis [2,3].

## Figures and Tables

**Table 1:** Overrepresentation of enriched Gene Ontology (GO) terms within genes co-segregating with gastroschisis.

Category	GO term	Count	*P* Values (FDR)
BP	Xenobiotic glucuronidation	8	7.99 × 10^−11^
BP	Negative regulation of glucuronosyltransferase activity	7	1.42 × 10^−9^
BP	Flavonoid glucuronidation	8	2.26 × 10^−7^
BP	Response to hormone	30	1.65 × 10^−4^
BP	Response to chemical	75	3.9 × 10^−4^
BP	Regulation of fatty acid metabolic process	9	7.61 × 10^−4^
BP	Retinoic acid metabolic process	4	2.05 × 10^−2^
CC	Integral component of membrane	70	4.95 × 10^−3^
CC	Membrane part	75	5.55 × 10^−3^
CC	Endoplasmic reticulum part	23	8.92 × 10^−3^
CC	Cytoplasmic part	102	2.18 × 10^−2^
MF	Glucuronosyltransferase activity	8	1.58 × 10^−6^
MF	Retinoid binding	5	7.67 × 10^−3^
MF	Active transmembrane transporter activity	12	1.27 × 10^−2^
KP	Drug metabolism – other enzymes	9	1.32 × 10^−8^
KP	Pentose and glucuronate interconversions	8	2.83 × 10^−8^
KP	Retinol metabolism	9	1.1 × 10^−7^
KP	Steroid hormone biosynthesis	8	5.85 × 10^−7^
KP	Metabolism of xenobiotics by cytochrome P450	8	3.45 × 10^−6^

BP, biological process; CC, cellular component; MF, molecular function; KP, KEGG (Kyoto Encyclopedia of Genes and Genomes) pathway.

**Table 2:** Pathogenetic pathways from GO functional categories among selected genes co-segregating with gastroschisis *.

Pathogenetic Pathway	GO Terms	Genes Involved
Xenobiotic	Xenobiotic and flavonoid glucuronidation, retinol metabolism, negative regulation of fatty acid metabolic process, drug metabolic process, steroid hormone biosynthesis, negative regulation of cellular carbohydrate metabolic process, cellular response to xenobiotic stimulus, chemical carcinogenesis, negative regulation of lipid metabolic process, cellular hormone metabolic process	*UGT1A4, UGT1A3, UGT1A10, UGT1A8, UGT1A7, UGT1A6, UGT1A5, AOX1, UGT1A9*
Regulation of metabolic processes	Negative regulation of catalytic activity, negative regulation of molecular function, regulation of transferase activity, carboxylic acid metabolic process, response to growth factor, regulation of hydrolase activity, response to endogenous stimulus, detection of stimulus, regulation of protein modification process, folate biosynthesis	*UGT1A4, UGT1A3, PLEKHG4B, COL6A3, RASGRP1, HHIP, THBS1, ADCY9, PER2, KDM5A, SLC9A3, BCAS3, OR2C1, OR4C3, RPS3, OR13F1, OBSCN, UGT1A10, UGT1A8, UGT1A7, UGT1A6, PKD1, UGT1A9, RAPGEF1, FGFRL1, ZFHX3, MAP2K3, FGFR4, ITGA2, TLR8, OR10G4, ITIH3, NOTCH1, ALPP, PLOD1*
Regulation of cell adhesion	Cell-substrate adhesion, cell adhesion, regulation of cell junction assembly, negative regulation of anoikis, epidermis morphogenesis and development, epithelium development, cell-cell adhesion via plasma-membrane adhesion molecules, focal adhesion, wound healing	*COL6A3, RASGRP1, THBS1, CEACAM5, PTPRD, BCAS3, HHIP, PKD1, RAPGEF1, FGFRL1, ITGA2, F5, KLK14, EVPL, FGFR4, PLOD1, NOTCH1, MYBPC2*
Regulation of gene expression	Circadian regulation of gene expression, developmental biology, multi-organism reproductive process, negative regulation of nucleic acid-templated transcription	*PER2, ZNF717, KDM5A, ZFHX3, COL6A3, RASGRP1, KLK14, HIST1H2BB, CDYL, EVPL, FGFR4, ITGA2, NOTCH1*
Inflammatory response	Toll receptor signaling pathway, inflammatory response, regulation of cytokine biosynthetic process	*UBE2NL, MAP2K3, TLR8, RASGRP1, THBS1, AOX1, ITGA2, NOTCH1*
Regulation of vascular development	Circulatory system development, blood vessel development, hemostasis, blood coagulation	*HHIP, RASGRP1, THBS1, CEACAM5, ITGA2, F5, ITIH3, BCAS3, SGCD, PKD1, RAPGEF1, FGFRL1, NOTCH1*
Keratinization	Formation of the cornified envelope	*KLK14, EVPL*
Left-right symmetry	Left-right axis specification	*NOTCH1*
Epigenetic	Histone modification, chromatin organization, DNA methylation	*PER2, KDM5A, HIST1H2BB, CDYL*
Ubiquitination	Protein ubiquitination	*UBE2NL, PER2, RPS3*
Regulation of protein synthesis	Protein-containing complex assembly	*RASGRP1, RPS3, PDE4DIP, FGFRL1, HIST1H2BB, F5*

* Manually curated pathogenetic pathways based on related GO functional categories according to ToppGene [8] from likely pathogenic genes co-segregating in the family with recurrence for gastroschisis (*SPATA17, PDE4DIP, CFAP65, ALPP, ZNF717, OR4C3, MAP2K3, TLR8, UBE2NL, COL6A3, FGFRL1, HHIP*, *SGCD*, *RAPGEF1, PKD1*, *ZFHX3*, *BCAS3*, *EVPL*, *CEACAM5, KLK14, PLOD1, UGT1A3, UGT1A4, UGT1A5, UGT1A6, UGT1A7, UGT1A8, UGT1A9, UGT1A10, AOX1, NOTCH1, HIST1H2BB, RPS3, THBS1, ADCY9, FGFR4, OR10G4, OR2C1, OR13F1*, *ITIH3, PLEKHG4B*, *SLC9A3*, *ITGA2*, *PER2*, *PTPRD*, *CDYL, KDM5A*, *RASGRP1*, *MYBPC2*, *F5*, and *OBSCN)*.

## Supplementary Materials

Supplementary materials can be found at https://www.mdpi.com/1422-0067/20/9/2295/s1.

## Abbreviations

*ADCY9*	Adenylate cyclase 9 gene
*ALPP*	Alkaline phosphatase placental gene
*AOX1*	Aldehyde oxidase gene
*BCAS3*	Microtubule associated cell migration factor gene
BP	Biological process
CC	Cellular component
*CDYL*	Chromodomain Y like gene
*CEACAM5*	Carcinoembryonic antigen related cell adhesion molecule gene
*CFAP65*	Cilia and flagella associated protein 65 gene
*COL6A3*	Collagen type VI alpha 3 chain gene
*CROCC*	Ciliary rootlet coiled-coil, rootletin gene
*DPYD*	Dihydropyrimidine dehydrogenase gene
*EVPL*	Envoplakin gene
*F5*	Coagulation factor V gene
FDR	False discovery rate
*FGFR4*	Fibroblast growth factor receptor 4 gene
*FGFRL1*	Fibroblast growth factor receptor like 1 gene
GO	Gene ontology
*HHIP*	Hedgehog interacting protein gene
*HIST1H2BB*	Histone cluster 1 H2B family member B gene
*IFT140*	Intraflagellar transport 140 gene
*ITGA2*	Integrin subunit alpha 2 gene
*ITIH3*	Inter-alpha-trypsin inhibitor heavy chain 3 gene
*KDM5A*	Lysine demethylase 5A gene
KP	Kegg pathway
*KLK14*	Kallikrein related peptidase 14 gene
*MAP2K3*	Mitogen-activated protein kinase kinase 3 gene
MF	Molecular function
*MYBPC2*	Myosin binding protein C, fast type gene
*NOTCH1*	Notch 1 gene
*OBSCN*	Obscurin gene
*OR2C1*	Olfactory receptor family 2 subfamily C member 1 gene
*OR4C3*	Olfactory receptor family 4 subfamily C member 3 gene
*OR10G4*	Olfactory receptor family 10 subfamily G member 4 gene
*OR13F1*	Olfactory receptor family 13 subfamily F member 1 gene
*PDE4DIP*	Phosphodiesterase 4D interacting protein gene
*PER2*	Period circadian regulator 2 gene
*PKD1*	Polycystin 1 gene
*PLEKHG4B*	Pleckstrin homology and RhoGEF domain containing G4B gene
*PLOD1*	Procollagen-lysine, 2-oxoglutarate 5-dioxygenase 1 gene
*PTPRD*	Protein tyrosine phosphatase receptor type D gene
*RASGRP1*	RAS Guanyl releasing protein 1 gene
*RAPGEF1*	Rap guanine nucleotide exchange factor 1 gene
*RPS3*	Ribosomal protein S3 gene
*SGCD*	Sarcoglycan delta gene
*SLC9A3*	Solute carrier family 9 member A3 gene
*SPATA17*	Spermatogenesis associated 17 gene
SVS	SNP and variation suite
*THBS1*	Thrombospondin 1 gene
*TLR8*	Toll like receptor 8 gene
*UBE2NL*	Ubiquitin conjugating enzyme E2 N like gene
*UGT1A3*	UDP Glucuronosyltransferase family 1 member A3 gene
*UGT1A4*	UDP Glucuronosyltransferase family 1 member A4 gene
*UGT1A5*	UDP Glucuronosyltransferase family 1 member A5 gene
*UGT1A6*	UDP Glucuronosyltransferase family 1 member A6 gene
*UGT1A7*	UDP Glucuronosyltransferase family 1 member A7 gene
*UGT1A8*	UDP Glucuronosyltransferase family 1 member A8 gene
*UGT1A9*	UDP Glucuronosyltransferase family 1 member A9 gene
*UGT1A10*	UDP Glucuronosyltransferase family 1 member A10 gene
WD	Week of development
WES	Whole exome sequence
*ZFHX3*	Zinc finger homeobox 3 gene
*ZNF717*	Zinc finger protein 717 gene
